# Exploring traditional Chinese medicine to enhance the efficacy and reduce toxicity of immunotherapy for non-small cell lung cancer from the perspective of “spleen deficiency”: a trial protocol for mechanism and clinical evidence

**DOI:** 10.3389/fonc.2026.1926389

**Published:** 2026-09-03

**Authors:** Cunya Li, Xiaonan Zhang, Shujuan Fu, Pantong Wu, Zhiying Wang, Zhixian Zhong, Yi Zhong

**Affiliations:** 1 Oncology Department, Shanghai TCM-Intergrated Hospital Shanghai University of Traditional Chinese Medicine, Shanghai, China; 2 Shanghai University of Traditional Chinese Medicine, Shanghai, China; 3 Department of Oncology I, Yueyang Hospital of Integrated Traditional Chinese and Western Medicine, Shanghai University of Traditional Chinese Medicine, Shanghai, China; 4 Department of Oncology, East Hospital Affiliated to Tongji University, School of Medicine, Tongji University, Shanghai, China

**Keywords:** immunotherapy, Jianpi Guchang prescription, NSCLC, spleen deficiency type, traditional Chinese medicine (TCM)

## Abstract

**Introduction:**

Although immune checkpoint inhibitors (ICIs) have changed the treatment landscape of advanced non-small cell lung cancer (NSCLC), their low response rates and immune-related adverse reactions (irAEs) remain serious challenges. Traditional Chinese medicine believes that “spleen deficiency” is the core pathogenesis of advanced lung cancer, and its corresponding immune suppression and metabolic disorders may be related to the efficacy of ICIs. This paper aims to explore the clinical application status and potential mechanisms of traditional Chinese medicine combined with ICIs based on the “strengthening the spleen”. Literature analysis shows that traditional Chinese medicines for invigorating spleen have shown positive trends in improving patients ‘quality of life and reducing irAEs. Their mechanism of action may involve pathways such as regulating the “gut-lung axis” and reshaping the tumor immune microenvironment. However, this field still faces challenges such as the lack of high-quality randomized controlled trials, inconsistent dialectical standards, and unclear molecular mechanisms. Accordingly, we designed the present trial to explore the therapeutic efficacy and safety profile of JPGC combined with ICIs in patients with NSCLC.

**Methods/design:**

The evaluation design of the JPGC clinical trial was a randomized, double-blind, placebo-controlled study. The sample size is 56, and there are 28 participants in the test group and control group respectively. The test group received JPGC, and the control group received JPGC placebo. There is a course of treatment every 21 days for a total of 4 courses. The main outcomes include 6-month PFS rate, secondary outcomes include PFS, exploratory study endpoints include TCM syndrome integral, EORTC QLQ-C30, ECOG score, intestinal barrier, intestinal flora, inflammatory cytokines, T cell subsets, etc.

**Discussion:**

We predict that by repairing the intestinal barrier and adjusting the stability of intestinal flora to enhance the efficacy of immunotherapy for non-small cell lung cancer, non-small cell lung cancer patients will improve spleen deficiency and quality of life through JPGC. This study used TCM syndrome analysis, laboratory tests, imaging examinations and other indicators to evaluate the clinical efficacy and safety of JPGC. Fecal metabolomic analyses will be implemented in trial subjects to explore the latent synergistic mechanisms associated with JPGC intervention. This is a traditional Chinese medicine trial to observe and treat advanced NSCLC characterized by spleen deficiency syndrome by directly regulating intestinal flora. If successful, this discovery will have value in antitumor therapies and drug development, especially in the “immune era.”

**Clinical Trial Registration:**

https://itmctr.ccebtcm.org.cn, identifier ITMCTR2025002090.

## 1 Introduction

Lung cancer ranks first in the world’s cancer cause (1), of which non-small cell lung cancer (NSCLC) accounts for approximately 85% (2). Immunotherapy with PD-1/PD-L1 inhibitors as the core has been extended from advanced to perioperative periods, bringing long-term survival benefits by reshaping tumor-immune interactions (3), but its clinical limitations are significant: it is difficult to accurately screen beneficiaries, and the suppressed state of the tumor immune microenvironment (TME) and brain metastases limit efficacy (4, 5); immune-related adverse reactions (irAEs) are mainly organ-specific inflammation, and combined treatment adds risks (6, 7); Drug resistance involves multiple mechanisms such as primary, acquired and physical barriers, and some patients even experience hyper progression (8–10). For stage IIIB-IV NSCLC, although ICIs are standard treatment, the single-agent response rate is still limited, and irAEs are common.

Traditional Chinese medicine can increase efficiency, attenuate toxicity, delay drug resistance and improve quality of life (11, 12), It can enhance efficacy by regulating immunity and reshaping TME (13), reduce the incidence of irAEs (14), and use multi-target intervention to delay drug resistance (15). Traditional Chinese medicine regards the “spleen” as the foundation of the day after tomorrow. Patients with advanced lung cancer generally have the pathogenesis of “spleen qi deficiency”, which is manifested by fatigue, poor appetite, and loose stools. “Spleen deficiency” is closely related to low immune function, chronic inflammation and intestinal flora disorders (16–18), which are the key factors affecting the efficacy of ICIs. Jianpi Guchang prescription (The drug composition is detailed in **Table 1**) is a self-made clinical experience prescription based on the theory that “the lung and the large intestine are mutually exterior-interior”. Preliminary research by our research team found that JPGC intervention in chemotherapy patients can effectively resist the increase in intestinal mucosal permeability and intestinal inflammation caused by chemotherapy. Reaction, thereby protecting the intestinal barrier function, enhancing the body’s immunity, and promoting the recovery of intestinal flora diversity after chemotherapy (19, 20). Jianpi Guchang prescription maintains the steady state of intestinal flora and protects the intestinal barrier function by regulating the metabolism of short-chain fatty acids; at the same time, it promotes Foxp3 expression by down-regulating the activity of the PI3K/Akt signaling pathway, and indirectly regulates the tumor immune microenvironment, and ultimately exerts a tumor inhibitory effect (21). In the clinical application of patients with advanced metastatic colorectal cancer, this compound treatment has been shown to extend the median survival time of patients by 5.06 months and significantly improve the quality of life of patients. Therefore, the strategy of combining the principles of strengthening the spleen with ICIs has a solid theoretical basis and transformation potential. We designed this study to focus on the effectiveness and safety of JPGC combined with ICIs in the treatment of non-small cell lung cancer.

**Table 1:** Components of JPGC prescription.

Chinese name	Latin name	Source plant	Dosage (g)
Huang Qi	*Astragalus membranaceus*	The dried roots of Mongolian milkvetch Astragalus membranaceus (Fisch.) Bge. var. mongholicus (Bge.) Hsiao and membranous milkvetch Astragalus membranaceus (Fisch.)	30
Fu Ling	*Wolfiporia cocos*	Dried sclerotium of the polypore fungus Poria cocos (Schw.) Wolf.	10
Bai Zhu	*Atractylodes macrocephala*	Dried rhizome of Atractylodes macrocephala Koidz., a plant of the Asteraceae family	10
Dang Shen	*Codonopsis pilosula*	Dried root of Codonopsis pilosula (Franch.) Nannf., a Campanulaceae plant	10
Tu Fu Ling	*Smilax glabra*	Dried rhizomes of the Liliaceae plant Smilax glabra Roxb	10
Ba Qia	*Smilax china*	Dried rhizome of Smilax china L., a plant of the Liliaceae family	10
Teng Li Gen	*Actinidia arguta*	For the roots of the kiwifruit plant Actinidia chinensis Planch	10
Ba Yue Zha	*Holboellia angustifolia*	Fruit of the Akebia family plant Akebia trifoliata (Thunb) Koidz var. australis (Diels) Rehd. or three-leaf Akebia A. trifoliata (Thunb.) Koidz., five-leaf Akebia A. quinata (Thunb.) Decne	10
Ji Nei Jin	*Galli Gigerii Endothelium Corneum*	The inner wall of the dry gizzard of the domesticated chicken Gallus domesticus Brisson	5

## 2 Methods

### 2.1 Study design

Guided by the core theories of traditional Chinese medicine that “strengthening the body and treating cancer” and “the lung and the large intestine are mutually exterior-interior”, through Placebo-controlled randomized double-masked clinical investigation, we evaluated the efficacy of integrated traditional Chinese and Western medicine based on “Jianpi Guchang prescription” as the basic prescription in the treatment of spleen deficiency type stage IIIB-IV non-small cell lung cancer, prolonging patients ‘progression-free survival and improving their quality of life, obtaining high-level evidence-based medical evidence, and forming a standardized diagnosis and treatment plan that can be promoted.

### 2.2 Sample size calculation

The study estimated the sample size required for this study based on the main efficacy indicators. According to the preliminary clinical treatment results, the effective rate in the traditional Chinese medicine treatment group is 50%, and the effective rate in the control group is 10%. Assuming that the type I error probability of this study is α=0.05, and the power (1-β) = 90%. Therefore, substitute P1 = 50%, P2 = 10%, α=0.05, β=0.1, μ_α/2_ = 1.96, μ_β_=1.282 into the formula and n=23. Based on the above calculation, 23 cases are needed in each group. Taking into account the 20% sample loss rate, 28 cases should be recruited in each group, i.e., n1=n2 = 28, for a total of 56 cases in the two groups.


$$n=\frac{p_{1}\times (1-p_{1})+p_{2}\times (1-p_{2})}{{\left(p_{1}-p_{2}\right)}^{2}}\times {\left(\mu_{\alpha /2}+\mu_{\beta }\right)}^{2}$$

### 2.3 Recruitment

**Figure 1:**
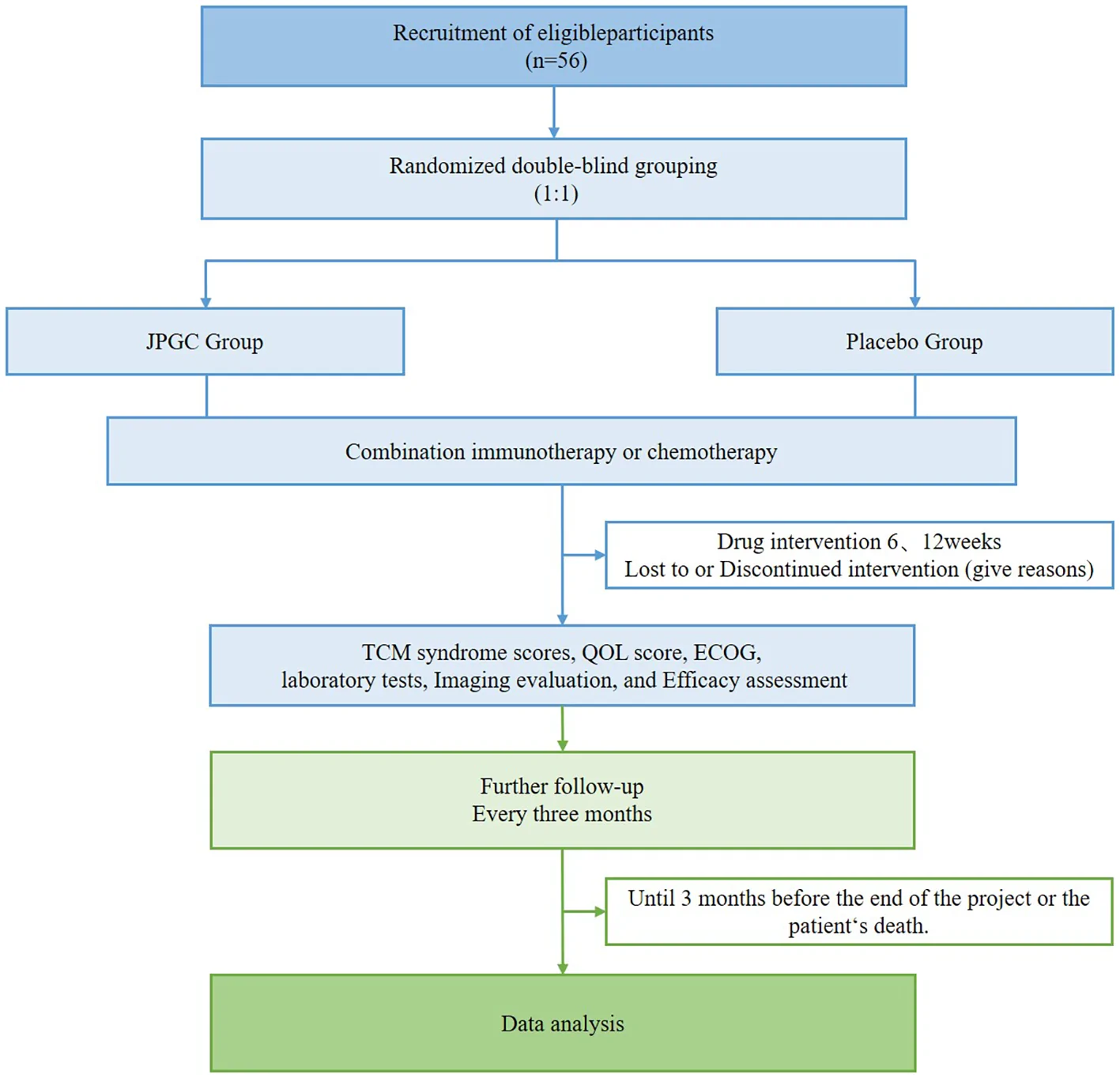
Participant flowchart.

The 56 cases in this study were all from advanced lung cancer patients who received immunotherapy at Shanghai TCM-Intergrated Hospital Shanghai University of Traditional Chinese Medicine. All subjects participating in this trial shall provide written informed consent prior to enrollment. The trial workflow is illustrated in **Figure 1**. Subject recruitment remains ongoing as of the date of submission. The first subject was enrolled on June 25, 2026, and the trial is projected to conclude in October 2027.

## 3 Participants selection

### 3.1 Diagnostic criteria

#### 3.1.1 Modern medical diagnosis

Refer to the “Guidelines for the Diagnosis and Treatment of Non-Small Cell Lung Cancer” formulated by CSCO in 2024; TNM pathological staging (pTNM) adopts the 8th edition of AJCC/UICC.

#### 3.1.2 TCM diagnosis

Two deputy chief physicians independently conducted syndrome differentiation according to the “Clinical Research Guidelines for the Treatment of Spleen Syndrome with Traditional Chinese Medicine” to determine whether the diagnostic criteria for spleen deficiency syndrome were met. Clinical manifestations must have more than 2 or at least 2 spleen main symptoms, or 1 spleen main syndrome and 2 spleen secondary syndromes. When the conclusions of the two are inconsistent, the final syndrome results will be reviewed by a more senior chief physician of traditional Chinese medicine.

##### 3.1.2.1 Main symptoms

① Reduced appetite or poor appetite; ② abnormal stool (loose, rotten, dry first and then thin, etc.); ③ abdominal distension after eating or abdominal distension in the afternoon.

##### 3.1.2.2 Secondary symptoms

④ The mouth is light but not thirsty; likes hot drinks; ⑤ The mouth is full of clear saliva; ⑥ Continuous abdominal pain, or likes warm and pressed pressure, or reduces when you get food, or it occurs when you feel tired; ⑦ Nausea and vomiting; ⑧ Stomachiness; ⑨ Bowersounds; ⑩ Weight loss or puffy; pale complexion; pale lips; Shortness of breath; Weak bowel movement; leucorrhoea; clear and thin urine; Excessive phlegm; Insomnia and insomnia.

Tongue pulse: pale tongue, teeth marks on the sides, white and thick greasy coating; pulse number.

Anyone who meets any 2 of the above main symptoms ① to ③, or any 1 of ① to ③ plus any 2 of ④ to, and satisfies the spleen deficiency tongue pulse, can be identified as spleen deficiency syndrome.

### 3.2 Inclusion criteria

1. A patient with pathologically confirmed non-small cell lung cancer and stage IIIB-IV who did not meet the surgical indications according to UICC version 8;
2. It conforms to traditional Chinese medicine syndrome differentiation and is classified as spleen deficiency syndrome;
3. Willing to receive immunotherapy;
4. No antibiotics, systemic corticosteroids (or prednisone doses less than 10mg/day) or other immunosuppressants were used within 1 month before enrollment;
5. No inflammatory bowel disease, radiation enteritis, rheumatic immunity and other diseases;
6. No other serious complications;
7. Aged between 18 and 80 years inclusive, male or female subjects;
8. ECOG ≤ 2, estimated survival time ≥3 months;
9. Baseline laboratory tests met the following criteria: neutrophils ≥1.5×10^9/L, platelets ≥80×10^9/L, hemoglobin ≥ 90 g/L, Serum alanine aminotransferase (ALT) and aspartate aminotransferase (AST) ≤2.5 times the upper limit of normal (if AST is allowed when liver metastasis is present, ALT ≤ 5 times the upper limit of normal), bilirubin ≤1.5 times the upper limit of normal, and creatinine ≤1.5 times the upper limit of norma, serum creatinine ≤ULN to 1.5 times, INR ≤ 1.5 or prothrombin time (PT) extension no more than 3 seconds in normal control.

### 3.3 Exclusion criteria

1. Those with severe primary diseases such as severe cardiovascular and cerebrovascular diseases, as well as mental illness;
2. Poor compliance and incomplete data during clinical research;
3. The diagnosis is unclear;
4. Subjects who cannot perform the provisions of this experimental protocol;
5. Subjects whose observation items are incomplete and cannot evaluate the test effect and side effects;
6. Those who have participated in other clinical trials in the past 3 months;
7. Patients who are pregnant or breastfeeding;
8. Those who have received other immune-related therapies or other therapies that may affect immune function;
9. During the medication process, those who suffer from other diseases due to non-drug factors and cannot continue to complete treatment.

### 3.4 Elimination criteria

1. Those who do not meet the inclusion criteria and mistakenly accept them;
2. Those who meet the inclusion criteria but fail to implement the trial plan after inclusion.

The reasons should be explained for excluded cases, and the case report form (CRF) form should be retained for future reference.

### 3.5 Drop-out criteria

1. Abnormal function of important organs of the subject during the clinical trial;
2. The subject experienced severe drug allergic reactions during the clinical trial;
3. Serious adverse reactions require discontinuation of trial drug treatment;
4. Patients cannot tolerate adverse drug reactions;
5. Poor patient compliance during the clinical study affects the effectiveness and safety evaluation, and subjects cannot be completed.

### 3.6 Discontinuation criteria and voluntary withdrawal of participants

1. If a serious adverse event occurs, the doctor judges whether the clinical trial of the case should be stopped.
2. If serious deviations occur due to poor compliance during the implementation of clinical trial protocols, it is difficult to evaluate the drug effect.
3. Subjects who are unwilling or impossible to continue the clinical study for whatever reason and are discontinued by submitting a request to the competent doctor to withdraw from the study.

### 3.7 Randomization, allocation, and blinding

Randomized method: This study adopted a completely randomized, double-blind, placebo-controlled design. A total of 56 eligible participants were enrolled and randomly assigned at a 1:1 ratio to the experimental group and control group (28 cases per group) without stratification. The complete random allocation sequence was independently generated using SPSS 26.0 statistical software. All study drugs were uniformly coded according to the finalized random sequence, with unique drug numbers clearly marked on the outer packaging. The random sequence and allocation list were securely managed by an independent dedicated researcher who was not involved in participant recruitment, clinical assessment, sample testing, or data analysis, ensuring strict allocation concealment throughout the trial.

Blinding method: A four-level rigorous blinding strategy was implemented throughout the entire trial, with participants, clinical investigators, imaging/outcome assessors, and statistical analysts remaining fully blinded. Formal unblinding was only performed after data entry, data verification and database lock. To quantitatively assess blinding quality, a pre-specified post-trial blinding validity evaluation was conducted. Before official unblinding, all participants and attending investigators completed a standardized blinding guess questionnaire, and guessing accuracy was calculated to verify blinding efficacy and control detection bias. Emergency unblinding was only permitted for life-threatening adverse events requiring urgent clinical management, and was performed only by the principal investigator. All unblinding events were fully documented. Participants with emergency unblinding were unblinded for clinical management but remained in safety follow-up and safety data analysis. All participants provided written informed consent before enrolment.

### 3.8 Intervention method

1. Treatment group: Jianpi Guchang granules, 1 bag each time, twice a day. Combined with immunotherapy (a. Define and stratify according to PD-L1 tumor proportion score and EGFR/ALK/KRAS/BRAF status, driver gene negative, any PD-L1 level: platinum-containing chemotherapy combined with immunotherapy is an important first-line option. When PD-L1 TPS>1% subjects, combined PD-1/PD-L1 immunotherapy is allowed. b. Choose a chemotherapy regimen that complies with guideline recommendations). There is a course of treatment every 21 days, and there are a total of 4 courses of treatment.
2. Control group: The control group received oral granules at 1/10 of the original dose of the treatment group, and other treatment options were the same as those in the treatment group. Every 21 days is a course of treatment, and a total of 4 courses of treatment are provided.

Granules:The granules and placebo used in the trial were produced by Shanghai Wanshicheng Pharmaceutical Co., Ltd.

**Table 2** presents the timeline for subject screening, trial interventions and study assessments.

**Table 2:** Trial timeline.

Visit	Screening	Drug intervention period (weeks)				After drug withdrawal	Further follow-up (Every three months until 3 months before the end of the project or the patient's death)
	0d	3	6	9	12	3 months	
Informed consent form	×						
Demographic information	×						
Inclusion and exclusion criteria	×						
General information	×						
Vital signs	×						
History of NSCLC and its treatment	×						
History of other diseases and their treatments	×						
TCM syndrome score	×	×	×	×	×		
ECOG score	×	×	×	×	×		
EORTC QLQ-C30	×	×	×	×	×		
Routine blood tests	×		×		×		
Liver function	×		×		×		
Renal function	×		×		×		
Serum and stool samples collection	×		×		×		
Intestinal barrier function	×		×		×		
Gut flora	×		×		×		
Inflammatory cytokines	×		×		×		
T cell subsets	×		×		×		
Imaging evaluation (RESIST1.1)	×		×		×	×	×
Adverse events		×	×	×	×	×	×
Release drugs/placebo	×	×	×	×			
Retrieve empty packages		×	×	×	×		
Drug combination	×	×	×	×	×	×	×
Survival					×	×	×

## 4 Outcome evaluation

### 4.1 Primary outcome

6-month PFS rate: the proportion of all randomized participants who are free of disease progression (assessed per iRECIST criteria) and free from all-cause death at the 6-month time-point from randomization.

### 4.2 Secondary outcome

Progression-free survival (PFS), from the start of randomization to the time when the tumor progresses.

### 4.3 Exploratory study endpoints

Including TCM syndrome integral, EORTC QLQ-C30, ECOG score, intestinal barrier, intestinal flora, inflammatory cytokines, T cell subsets, etc.

TCM syndrome integral: TCM syndrome integral record time refer to evaluation time point in **Table 2**.

TCM Syndrome Points:

The severity of primary symptoms and secondary symptoms was divided into 0, I, II and III according to the single scoring standard of primary symptoms and secondary symptoms. Grade 0: asymptomatic, 0 points; Grade I: mild symptoms, not affecting daily life and work, 1 point; Grade II: moderate symptoms, partially affecting daily life and work, 2 points; Grade III: severe symptoms, affecting daily life, difficult to persist in work, 3 points.

The evaluation of syndrome efficacy includes changes in main symptoms, secondary symptoms, signs, and tongue images. It is calculated using nimodipine method. The efficacy index = [(pre-treatment points subtract post-treatment points)/pre-treatment points]×100%, which is divided into 4 levels: clinical recovery, effective, and ineffective.

1. Clinical recovery: The main symptoms, secondary symptoms, and signs disappeared or basically disappeared, the tongue picture basically returned to normal, and the efficacy index was ≥95%;
2. Effective effect: The main symptoms, secondary symptoms, signs, and tongue symptoms have been significantly improved, with 70%≤ efficacy index <95%;
3. Effective: The main symptoms, secondary symptoms, signs, and tongue symptoms have improved, 30%≤ efficacy index<70%;
4. Ineffectiveness: The main symptoms, secondary symptoms, signs, and tongue manifestations have not been significantly improved or even worsened, and the efficacy index is<30%.

Quality of life QOL score and weight monitoring: application of EORTC QLQ-C30 scale.

A standardized PRO management and compliance monitoring plan is preset for the study: questionnaires are filled out independently at baseline, every 2 treatment cycles, and on site at the end of treatment. The investigators only provide process answer questions and do not induce answers. The questionnaire completion rate, missing item rate and compliance were monitored throughout the process; cases with low compliance were recorded, and the robustness of the results was verified through sensitivity analysis.

Patient performance status score (ECOG score): It was determined according to the ECOG performance status score standard. The ECOG score of enrolled patients was 0–2 points.

Efficacy assessment (RESIST1.1 criteria)

1. Tumor response assessment was performed simultaneously with RECIST 1.1 and iRECIST; preset imaging scanning time points: baseline, every 6 weeks, end of treatment, and follow-up period;
2. Set up a centralized image review at a third-party center, with two blinded radiology experts independently reviewing the images, and the differences will be decided by a third expert;
3. The rules for handling pseudo-progression are predefined: When immune-related pseudo-progression is suspected, confirmation imaging review will be performed 4–8 weeks later; iUPD will not be judged as PD under the iRECIST criteria, and subjects will continue to be allowed to continue the trial medication; disease progression will be judged only if iCPD is confirmed.

### 4.4 Microbiome and metabolomic analyses

Pre-specified mechanistic hypothesis: The TCM intervention modifies gut microbial community structure and host metabolic profiles in patients with advanced NSCLC, remodels the immune microenvironment via the gut-lung axis, and synergistically enhances anti-tumor therapeutic effects.

Sample collection and handling SOP: Fecal samples are collected at baseline and after two treatment cycles. Fresh fecal samples are immediately stored at −80 °C and shipped to the third-party laboratory on dry ice. Repeated freeze-thaw cycles are prohibited. Timestamps for sample collection, storage and transportation are fully documented.

Quality control (QC): For microbiome assays, DNA concentration and integrity are used for sample qualification; samples with severe DNA degradation or insufficient concentration are excluded. Negative and positive controls are included for sequencing. For metabolomics, pooled QC samples are interspersed among study samples to monitor instrument stability; ion features with excessive signal variation are filtered out. All QC thresholds are predefined and implemented by the contracted laboratory.

Statistical workflows: Alpha- and beta-diversity and differential taxa analyses are performed for microbiome data. Differential metabolite screening is conducted for metabolomics data. The Benjamini-Hochberg procedure is applied for FDR correction to control false-positive rates. Correlation analyses are performed to explore associations among microbes, metabolites and clinical endpoints. Analytical scripts are pre-defined; post-hoc modification by the testing vendor is not permitted. Raw datasets and QC reports are fully archived.

### 4.5 Safety observations

General vital signs, laboratory test results (including liver function and renal function tests) will be recorded before enrollment and during each treatment period (visits1,2, 3 and 4). Any possible adverse events (AEs) that occurred during all treatment periods were recorded. If the subject has abnormalities in indicators after treatment and no abnormalities before enrollment, regular monitoring will be performed until the indicators return to normal; all adverse events will be followed up until complete resolution.

### 4.6 Research implementation and safety monitoring

Composition of DSMB: 3 independent external experts, including 1 clinical oncology expert, 1 clinical statistics expert, and 1 pharmacology/safety expert; all members have no conflicts of interest with this study and will not participate in enrollment and clinical execution.

2) Interim analysis plan: One interim analysis is preset. The interim analysis will only be performed by independent statisticians, and the DSMB will review the safety and effectiveness data.

3) Toxicity evaluation uses CTCAEv5.0 uniformly, and all AE/SAE are graded and recorded according to this standard.

4) Preset stopping rules: including ① serious unacceptable safety risks; ② interim analysis shows that the benefit of the trial group has indeed reached the preset termination limit; ③ enrollment efficiency is seriously insufficient; the sponsor will decide whether to terminate the trial after the DSMB gives recommendations.

### 4.7 Trial quality control

All study personnel (including site investigators and study drug pharmacists) shall complete standardized trial-specific training prior to study initiation. Investigators shall complete case report forms (CRFs) fully in compliance with the study guidelines; CRFs constitute immutable source documents. All laboratory and imaging test results generated during the trial must be documented, with original or duplicate reports appended to the corresponding CRFs. Any laboratory parameters markedly outside clinical reference ranges shall be verified and annotated with explanatory notes by the study physician. Clinical monitors conduct routine on-site monitoring visits to investigative sites, perform random source data verification, host periodic thematic meetings, and deliver prompt feedback through monitoring reports and project synopses to sustain continuous improvement of trial quality.

Investigators will truthfully fill out the case report form, and inspectors will regularly check the data. The original record should be kept clearly visible during modifications, and the corrections should be signed and dated by the researcher. The case report form is reviewed by the supervisor and submitted to the clinical trial data manager. Transfers between researchers, supervisors and data managers will be specially recorded. Prior to data entry, the data manager shall conduct secondary data validation. All queries and corresponding resolutions shall be archived as query forms for retrospective review.

### 4.8 Data management

After the completion of the trial, the original data and collected samples shall be retained for a minimum of 5 years. All activities including trial safety monitoring, verification of the accuracy of subject information, protection of subjects’ privacy, and assessments concerning amendments to the study protocol or trial termination shall be directly undertaken by the trial research team.

### 4.9 Subject follow-up

The main forms of follow-up are telephone follow-up and outpatient follow-up. Patients are instructed to follow up one week after discharge. If the patient does not go to the outpatient follow-up, telephone follow-up will be changed to once every 3 months, and call records will be made. The survival period is calculated from the day of the first immunotherapy administration to the time of death recorded in the medical record or the patient’s death. The difference between the two times is the survival period; the last follow-up time for surviving patients and patients lost to follow-up is recorded as the censored time, and the survival time is expressed in months. The project conducted the last telephone follow-up visit for all enrolled patients as of the first 3 months.

### 4.10 Statistical analysis plan

Data processing was carried out with SPSS26.0 statistical software. The measurement data gives the mean, standard deviation, minimum, maximum, median, etc., which are expressed as mean ± standard deviation; the counting data gives the frequency distribution and corresponding percentages. T-test was used to compare data groups with normal distribution measurement data; rank-sum test was used for non-normal distribution measurement data, chi-square test was used for counting data, qualitative variables and correlation. Log-rank test was used for survival time and survival rate after treatment. Survival analysis was carried out using Kaplan-Meier method. Using α=0.05 as the test criterion, P<0.05 as the difference is statistically significant.

Information on the start and end times and treatment courses of antibiotics, proton pump inhibitors (PPIs), and probiotics during the trial will be collected, and key dietary covariates will be collected; subjects will not be forced to withdraw from the study due to the use of the above drugs. The preset sensitivity analysis included: excluding subjects who used antibiotics during follow-up; incorporating PPI, probiotics and dietary covariates into the model for correction to evaluate the robustness of the primary endpoint and microbiome and metabolome analysis results.

## 5 Discussion

### 5.1 Relationship between spleen deficiency syndrome and tumor immune microenvironment

The traditional Chinese medicine theory of “the spleen grows from the lung “ provides a framework for explaining the core pathogenesis of advanced NSCLC. Such patients have deficiency of vital qi, lack of healthy circulation of the spleen, no source of blood and blood biochemistry, and mutual phlegm and blood stasis, forming a pathological pattern with spleen deficiency as the foundation and phlegm and blood stasis as the target. Clinical data shows that the detection rate of spleen deficiency syndrome in patients with advanced NSCLC is high (22), and its severity is positively correlated with PS score.

From the perspective of modern medicine, there are multiple intersections between the biological essence of “spleen deficiency” and the tumor immunosuppressive microenvironment (23). Under the condition of spleen deficiency, the body’s immune homeostasis is significantly disrupted, which not only leads to a decline in the function of anti-tumor immune cells such as effector T cells and NK cells, but also promotes the large enrichment of immunosuppressive cells such as tumor-associated macrophages (TAM) and myeloid suppressor cells (MDSC) (24, 25), at the same time, it is accompanied by problems such as disorder of the inflammatory factor network, abnormal energy metabolism, imbalance of intestinal flora, abnormal blood vessels in tumor tissue, and local hypoxia (26–28), Together create a malignant microenvironment that promotes tumor immune escape, and may also induce primary or secondary resistance to immune checkpoint inhibitors (ICIs) (11). Therefore, spleen deficiency syndrome integrates multi-dimensional pathological information such as organ function, immune status, metabolic level, and intestinal microecology, providing an overall classification framework that transcends a single biomarker (29). “Strengthening the spleen” can be redefined as: reducing the intensity of immunosuppression by improving the host’s immune chassis and inflammatory background, thereby creating a more favorable “soil” for immunotherapy, cell therapy or tumor vaccines. This is the unique contribution of traditional Chinese medicine in modern precision medicine.

### 5.2 Clinical exploration of strengthening spleen Chinese medicine combined with immunotherapy

Several mechanism reviews summarize the multi-target role of traditional Chinese medicine in lung cancer: inducing cell death, reversing drug resistance, inhibiting metastasis, regulating immune responses, etc., providing a docking biological interface for “enhancing the efficacy of immunotherapy” (26, 30–32).

More importantly, there have been systematic reviews and meta-analyses of animal experiments specifically targeting “traditional Chinese medicine as immunotherapy synergists”, suggesting that the combination of traditional Chinese medicine and PD-1/PD-L1 inhibitors can improve tumor control, prolong survival and regulate mechanism axes such as cytokines, tumor microenvironment and intestinal flora, but also emphasize heterogeneity and evidence quality limitations (16). In addition, the “spleen deficiency” classification may make up for the limitations of markers such as PD-L1 and TMB, and help identify specific groups of people who have had poor benefits from immunotherapy.

### 5.3 Discussion on the mechanism of action: cascade reactions from “intestinal flora” to “tumor microenvironment”

Current research suggests that traditional Chinese medicine for strengthening the spleen does not use a single target, but uses a cascade network of sensitization immunotherapy dominated by the “gut-lung axis”. The comprehensive hypothesis can be summarized as follows: the spleen strengthening strategy first corrects the intestinal ecological imbalance under the spleen deficiency state., and then through systemic immune remodeling, ultimately reverses the local immunosuppressive microenvironment (TME) of the tumor.

The central hinge of this cascade lies in regulating intestinal flora and repairing intestinal mucosal barrier. Animal models of spleen deficiency syndrome and tumor-bearing generally have flora imbalance and intestinal mucosal barrier damage, which are characterized by decreased diversity and reduction of beneficial bacteria such as mucophilic Akkermannia (33, 34). Si Jun Zi Decoction and other Jianpi prescriptions can effectively increase the abundance of short-chain fatty acid-producing bacteria (35), promote tight junction protein expression to repair barriers (36). The bacterial community transplantation experiment provides causal evidence: transplanting fecal bacteria from mice interfered with by Chinese medicine for strengthening the spleen to sterile or antibiotic-treated mice can partially replicate the immunosensitizing effect, strongly proving that bacterial community regulation is the key to the effectiveness of the spleen medicine (37). The repaired intestinal barrier reduces abnormal translocation of bacteria and metabolites, reduces the level of systemic inflammation, and lays the foundation for subsequent immune regulation.

On this basis, healthy bacteria and their metabolites affect systemic immunity through circulation, constituting a systemic effect link connecting the “intestines” and the “lungs”. After strengthening the spleen, the imbalance in the ratio of CD4/CD8 T cells in the spleen and peripheral blood was reversed, the proportion of immunosuppressive Tregs and MDSCs decreased, and the chronic inflammatory state related to spleen deficiency was alleviated (38). More critically, the combination of spleen strengthening and immune checkpoint inhibitors can synergistically reduce the expression of inhibitory receptors such as PD-1 and TIM-3 on tumor-infiltrating T cells and reverse T cell functional depletion (39). It shows that spleen strengthening treatment not only increases the number of immune cells, but also optimizes their functional quality.

The improvement of systemic immunity ultimately needs to be implemented in the reshaping of the tumor microenvironment. On the indirect route, effector T cells activated by the “gut-lung axis” can more effectively recruit and infiltrate tumor tissue; on the direct route, astragalus polysaccharide and other components can promote the maturation of dendritic cells to enhance T cell activation (40), Ginsenoside Rg3 inhibits the VEGF pathway to promote normalization of tumor blood vessels and improve immune cell transport and function (41). Preclinical studies have also shown that small molecule drugs can achieve anti-tumor immunity through the stability of immunoregulatory checkpoint proteins (42). Pharmacological studies also suggest that active ingredients derived from traditional Chinese medicine can regulate intestinal flora and intestinal barrier functions and interfere with the tumor immune microenvironment, achieving a synergistic effect with immunotherapy (43–45). However, most of the above direct effects are based on single-component *in vitro* models, and their contribution to the complex human environment still needs to be further confirmed. This study explores the relationship between the regulation of the intestinal microenvironment and clinical benefits of traditional Chinese medicine compound formula through microbiome, metabolome, cytokines and intestinal barrier indicators, but is not enough to clarify the exact molecular targets and causal regulatory pathways. With the development of computational pharmacology, open platforms such as GEMap, COIMMR, and GSFM provide new strategies for tumor immune drug mining. Targeting and drug activity analysis can be carried out based on gene necessity, herbal components-immune metabolism association, and transcriptome functional modules. The hypotheses generated in this study can also be further confirmed through *in vitro* and animal experiments such as computational pharmacology. Comprehensive judgment, the indirect systemic effect produced by regulating intestinal flora may be the main mode of action of spleen strengthening immunotherapy.

## 6 Conclusion

After more than 2000 years of practice, the role of traditional Chinese medicine in lung cancer and other tumors is shifting from experience assistance to an integrated strategy driven by “mechanism + evidence”. Existing research shows that it can induce tumor cell death, inhibit metastasis, regulate immunity, and synergize with modern treatments to enhance overall benefits (31). Under the framework of integrated treatment, traditional Chinese medicine can not only directly affect tumor biology, but also improve the effectiveness and tolerance of chemotherapy, targeting and immunotherapy through systemic regulation such as inflammation, immunity, metabolism and microecology (46).

Drug resistance to ICIs is the core bottleneck of current immunotherapy. The potential value of traditional Chinese medicine lies in multi-dimensional intervention of tumor immune microenvironment and intestinal flora with lower toxicity to achieve “efficiency enhancement + anti-drug resistance + attenuation” (11). Relevant evidence shows that intestinal flora also determines tumor progression, efficacy and toxicity spectrum; traditional Chinese medicine can reshape the structure and metabolites of the flora, improve the intestinal barrier, and regulate immunity, while the biotransformation of traditional Chinese medicine components by the flora can enhance its anti-tumor activity, forming a two-way action network (47).

“Improvement of syndromes” should be defined in modern research as a quantifiable composite endpoint, that is, systematic improvement in symptom clusters (fatigue, appetite, sleep, pain, mood), functional status, and treatment tolerance (46). Its biological basis can be connected by the tumor-associated inflammatory microenvironment: IL-6, IL-10, TGF-β, VEGF, MMPs, TAM, MDSCs, etc. can be mapped to the state evolution of “heat → phlegm → stasis → deficiency”, thereby advancing “syndrome improvement” from subjective experience to state regulation that can be verified by targets and pathways (48).

Despite promising prospects, development in this area still faces several challenges. First, the level of evidence in clinical research needs to be improved. At present, most of them are small sample or retrospective studies, lacking well-designed, multi-center large-scale randomized controlled trials to provide high-level evidence. Secondly, the standardization of TCM syndrome differentiation is insufficient. The diagnosis of “spleen deficiency” syndrome mainly relies on the subjective experience of the doctor, while the scoring scale is the subjective judgment of the patient. It lacks objective and quantitative biological indicators, making it difficult to compare between different studies. Finally, the research on the mechanism of action is still at the exploratory stage. The components of traditional Chinese medicine compound are complex, and their specific interactions with the immune checkpoint pathway, key pharmacodynamic substances and core targets need to be deeply analyzed. Therefore, study design should avoid remaining in subjective benefits. Taking “treatment of strengthening the spleen” as an example, in addition to perceptible improvements in appetite, fatigue, weight, etc., objective endpoints (adverse reactions, QOL scale, immune indicators, intestinal function indicators, imaging evaluation results) must be bound.

At present, anti-tumor research on traditional Chinese medicine has been accurately analyzed with the help of multivariate omics, single cell and other technologies. At the same time, the combination of traditional Chinese medicine and immune checkpoint inhibitors has become a research hotspot. However, more rigorous clinical trial verification is still needed to explore the establishment of biomarkers Guided treatment strategies, for example, based on the patient’s intestinal flora characteristics or immune-related molecular typing, screen the advantageous groups who are most likely to benefit from combined treatment of spleen strengthening, and ultimately achieve precise treatment integrating traditional Chinese and Western medicine. Promote it from experience to an important part of verifiable and scalable comprehensive cancer treatment, providing a new path to break through the bottleneck of NSCLC immunotherapy.

## Author’s Note

This clinical trial is currently active. The first subject was screened and enrolled on 25 June 2025, with anticipated study completion scheduled for October 2027.

## Data Availability

The original contributions presented in the study are included in the article/supplementary material. Further inquiries can be directed to the corresponding author.

## Glossary

AEs: Adverse Events
ALT: Alanine Aminotransferase
AST: Aspartate Aminotransferase
CR: Complete Response
CF: Case Report Form
DCR: Disease Control Rate
ECOG: Eastern Cooperative Oncology Group Performance Status
ERG: ETS-Related Gene
IL-10: Interleukin-10
IL-6: Interleukin-6
INR: International Normalized Ratio
irAEs: Immune-Related Adverse Events
MDSC: Myeloid-Derived Suppressor Cell
MMPs: Matrix Metalloproteinases
NSCLC: Non-Small Cell Lung Cancer
ORR: Objective Response Rate
OS: Overall Survival
PD-1: Programmed Death-1
PD-L1: Programmed Death-Ligand 1
PFS: Progression-Free Survival
PR: Partial Response
PT: Prothrombin Time
QOL: Quality of Life
SAE: Serious Adverse Event
SD: Stable Disease
TAM: Tumor-Associated Macrophage
TGF-β: Transforming Growth Factor-β
TMB: Tumor Mutational Burden
TME: Tumor Microenvironment
ULN: Upper Limit of Normal
VEGF: Vascular Endothelial Growth Factor.
